# Ultrasound-guided cervical selective nerve root block in the approach to cervical traumatic neuromas: a technical note

**DOI:** 10.1590/0100-3984.2021.0173

**Published:** 2022

**Authors:** Vinícius Neves Marcos, Marco Aurelio Vamondes Kulcsar, Ana Oliveira Hoff, Maria Cristina Chammas, Ricardo Miguel Costa de Freitas

**Affiliations:** 1 Instituto do Câncer do Estado de São Paulo (Icesp), São Paulo, SP, Brazil.; 2 Instituto de Radiologia do Hospital das Clínicas da Universidade de São Paulo (InRad/HC-FMUSP), São Paulo, SP, Brazil.

## INTRODUCTION

A cervical traumatic neuroma (CTN), defined as non-neoplastic hyperplasia of a damaged peripheral nerve^(1)^, may be misinterpreted as a metastatic lymph node on postoperative ultrasound^(2)^. Fine needle aspiration biopsy (FNAB) of a CTN is quite painful^(2)^, so much so that acute exacerbation of pain during needle penetration into the nodule has been used as a diagnostic criterion for neuroma^(3)^.

The purpose of this case series was to describe a novel technique to reduce pain during FNAB of CTNs, by using an ultrasound-guided cervical selective nerve root block (SNRB).

## PROCEDURE

Two patients with metastatic papillary thyroid carcinoma underwent total thyroidectomy and lateral neck dissection, after which they presented with cervical nodules on postoperative ultrasound. The nodules were not characteristic of lymph nodes and presented an intimate relationship with the C4 nerve root.

**Figure 1:**
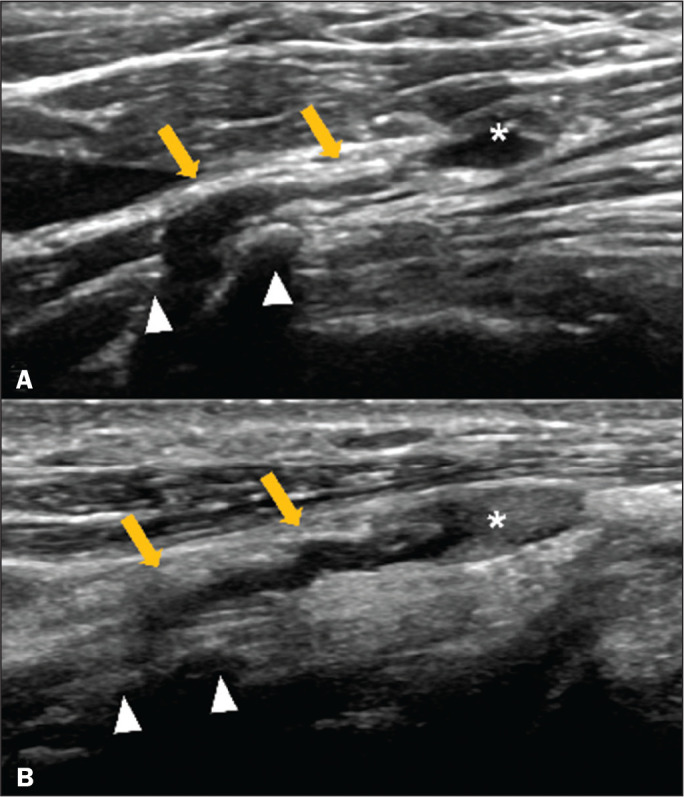
Ultrasound view at the C4 level in patients 1 (A) and 2 (B), each showing a fusiform neuroma (asterisks) in continuity with the nerve roots (arrows). The intervertebral foramen is located between the anterior and posterior tubercles of the transverse process (arrowheads).

The examinations were performed with a versatile ultrasound system (LOGIQ E9; GE Healthcare, Waukesha, WI, USA) equipped with a high-frequency linear transducer (13-15 MHz). The cervical nodules were identified at cervical level III, and continuity with the C4 nerve root was detected in both patients. The nerve root, located between the scalenus medius and longus capitis muscles, was followed down to its emergence between the anterior and posterior tubercles of the transverse process (Figure 1).

**Figure 2:**
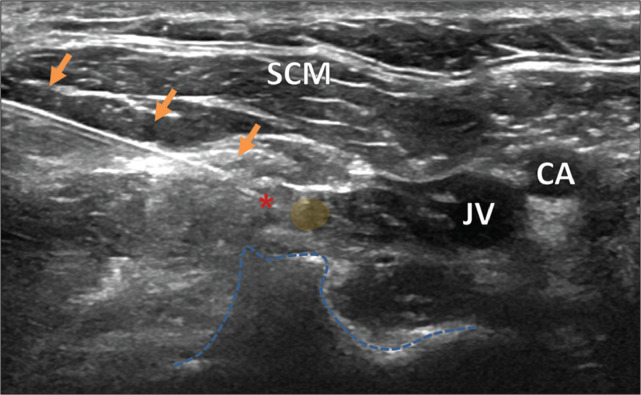
Ultrasound-guided cervical selective nerve block at the C4 level. The needle (arrows) is advanced caudally, at an angle of 45-60°, until its tip (asterisk) comes close to the C4 nerve root (yellow circle). The shape of the transverse process is delineated (blue line). The carotid artery (CA) and jugular vein (JV) are located medial to the needle track, whereas the sternocleidomastoid muscle (SCM) is located superficially.

After the initial ultrasound analysis, the skin was appropriately prepared with antiseptic, and the transducer was draped with a sterile cover. A cutaneous nerve block was performed with a local anesthetic (lidocaine 1%). A 22-G needle was guided percutaneously with the in-plane technique, at an angle of 45-60°, until reaching the C4 intervertebral foramen (Figure 2). After careful aspiration, 2 mL of lidocaine 1% were administered, under direct visualization by ultrasound, around the C4 root.

During the FNABs performed after the SNRB, there were no immediate complaints of pain. There were also no complications, and no additional therapy was immediately necessary. At one hour after the procedures, both patients reported mild pain (1/10 on a visual analogue scale) and were discharged with a prescription for oral analgesics. The cytological analysis revealed spindle cells, consistent with a diagnosis of neuroma, in both of the nodules biopsied.

## DISCUSSION

**Figure 3:**
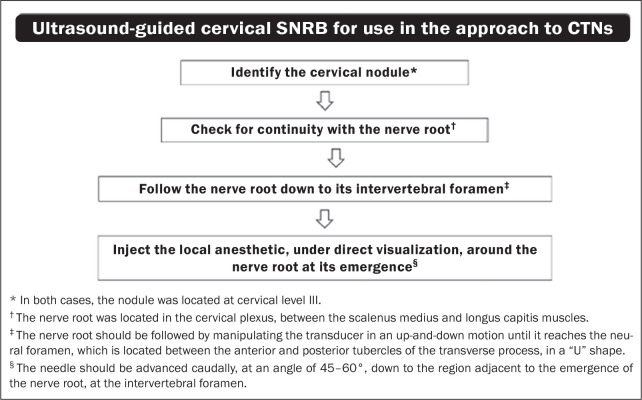
Algorithm summarizing the ultrasound-guided cervical SNRB technique used in the approach to CTNs.

This case series demonstrates a modified ultrasound-guided SNRB technique to use in the approach to CTNs, with efficient pain control during FNAB (summarized in Figure 3). To our knowledge, this technique has not previously been reported. It differs from conventional SNRB^(4-7)^ in that it is first necessary to identify the neuroma and its root, then to follow it down to its emergence at the vertebral foramen, ensuring the correct location for the block.

A potential complication of C4 root block is phrenic nerve palsy^(8)^. However, that complication was not observed in either of the cases presented here or in any of the reports of SNRB in the literature^(4-7)^. One reason for this may be the selective nature of the block, in which a small dose of anesthetic is used and is injected into only one nerve root, without blocking the other phrenic nerve roots^(4)^.

## CONCLUSION

In conclusion, the technique of using ultrasound-guided SNRB in the approach to CTNs, as described here, is safe and can be used in cases in which FNAB becomes necessary. The use of this technique can make that experience less traumatic for the patient.
